# Pleural Metastasis From Male Breast Cancer: A Case Report

**DOI:** 10.1002/dc.70049

**Published:** 2025-11-15

**Authors:** Patrizia Straccia, Esther Diana Rossi

**Affiliations:** ^1^ Division of Anatomic Pathology and Histology Catholic University of Sacred Heart, Foundation “Agostino Gemelli” University Hospital Rome Italy

**Keywords:** breast cancer, cytology, diagnosis, effusions, metastasis

## Abstract

Male breast cancer (MBC) is a rare disease accounting for less than 1% of breast cancers and for 0.11% of all male malignancies. Despite the fact that the epidemiologic, clinical, and therapeutic literature regarding female breast cancer is well documented, little is known about the features of male breast cancer. Here, we present a case of cytological pleural metastasis from ductal breast carcinoma in a 55‐year‐old man. Immunohistochemical staining showed that the tumor cells were positive for BerEp4, GATA‐3, AE1/AE3, CAM5.2, ER (70%), AR (10%), PR (10%), and ki67 (10%). In our experience, effusion cytology remains an accurate tool for the diagnosis of metastatic carcinomas.

## 1 Introduction

According to the literature, male breast cancer is a rare disease accounting for less than 1% of all breast cancers, and for 0.11% of all male malignancies [1, 2, 3, 4, 5, 6]. The incidence is changing over the last years [2, 3] even though there are few studies investigating the disease. Therefore, most available data come from observational retrospective investigations limited by small sample sizes, short follow‐up, and non‐population‐based design, with a consequent limitation in their interpretability.

Data from the literature has shown that the pathogenesis of male breast cancer is still unclear, although epidemiologic risk factors may include prostate cancer and associated endocrine therapy, gynecomastia, occupational hazards (e.g., exposure to electromagnetic fields, polycyclic aromatic hydrocarbons, high temperatures), dietary factors (e.g., meat intake) and alcohol intake [7].

It is well documented that BRCA1, BRCA2, and MMR gene mutations may play pivotal roles, as well as other genetic factors such as the AR gene, the CHEK2 gene, cytochrome P45017 (CYP17), the XXY karyotype (Klinefelter's syndrome), and the PTEN tumor suppressor gene associated with Cowden syndrome that might be involved. Comparing with the more common female breast cancer, the male counterparts have a later onset of disease and a more advanced stage [5, 6, 7, 8, 9].

Here we report an unusual case of cytological pleural metastasis from male breast cancer. We also include the results of a literature search performed for all cases of MBC with pleural effusion published so far.

## 2 Case Report

A 55‐year‐old man presented with pleural effusion. He had been treated 6 years before for ductal carcinoma of the breast with surgery (radical mastectomy of the left breast) followed by adjuvant chemotherapy (Halaven‐Eribulin), and radiation therapy (46 Gy). In October 2024, whole‐body positron emission tomography/computed tomography revealed a left pleural effusion.

Thoracentesis was carried out under computed tomography guidance by the surgeon. A pleural drainage was inserted to manage pleural effusion, under local anaesthesia and sterile conditions. No rapid on‐site evaluation to assess the adequacy of material was done. One pass or more was performed until macroscopically satisfactory material was harvested; an amount ranging from 1000 to 1500 mL of bloody fluid was drained and collected for cytology study.

The aspirate was processed using the ThinPrep method (ThinPrep 5000; Hologic Inc., Marlborough, MA). The material was fixed with the hemolytic and preservative solution Cytolyt after rinsing the needle in this solution. The cells were spun at 1500 rpm; then, the sediment was transferred in the Preservcyt solution to be processed with the T2000 automated processor according to the manufacturer's recommendation. The resulting slide was fixed in 95% ethanol and stained with the Papanicolaou method. Immunohistochemical staining was performed on the cell block, according to clinical protocols validated in our laboratory. Genetic tests demonstrated no mutations in the BRCA1 and BRCA2 genes.

## 3 Cytopathologic Findings

Abundant cellularity with groups of loosely cohesive malignant cells and individual scattered tumor cells can be observed. The neoplastic cells are small to medium‐sized with hyperchromatic, eccentric nuclei and fine to coarse chromatin. The cytoplasm can be basophilic, or finely vacuolated.

**FIGURE 1:**
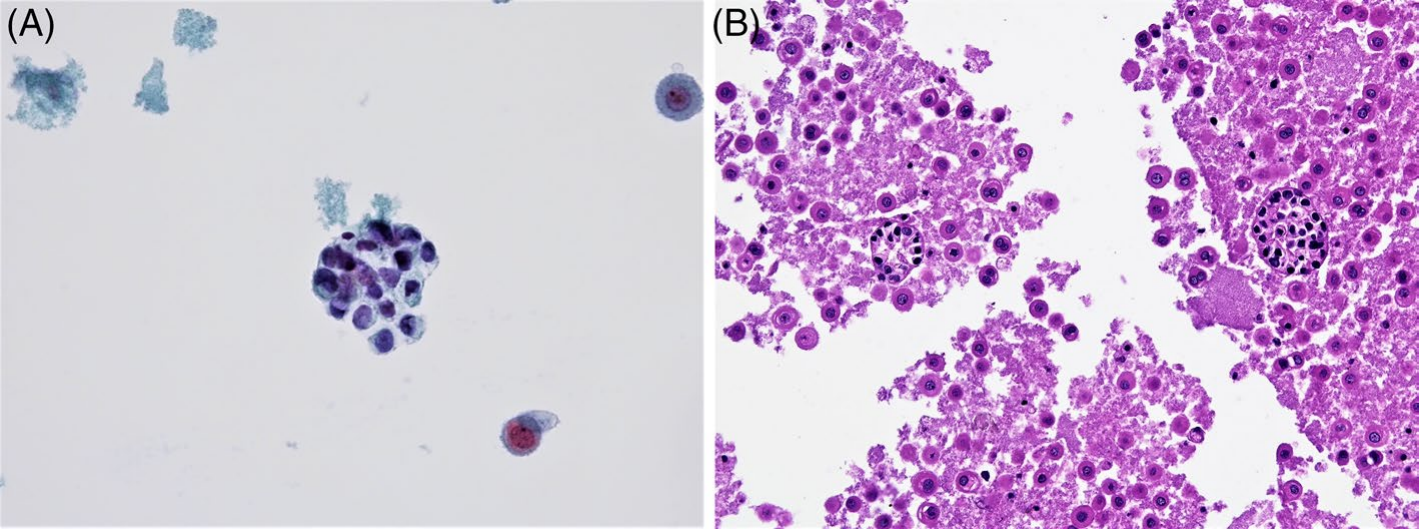
(A) A cluster of small to medium‐sized neoplastic cells with hyperchromatic nuclei (Papanicolau, original magnification 400×). (B) Cell block (H&E, original magnification 200×).

The malignant clusters show loss of polarity. In the background, individual tumor cells demonstrate malignant cytologic features including anisonucleosis, irregular nuclear borders, increased N/C ratios with small to prominent nucleoli (Figure 1A).

## 4 Immunohistochemistry

**FIGURE 2:**
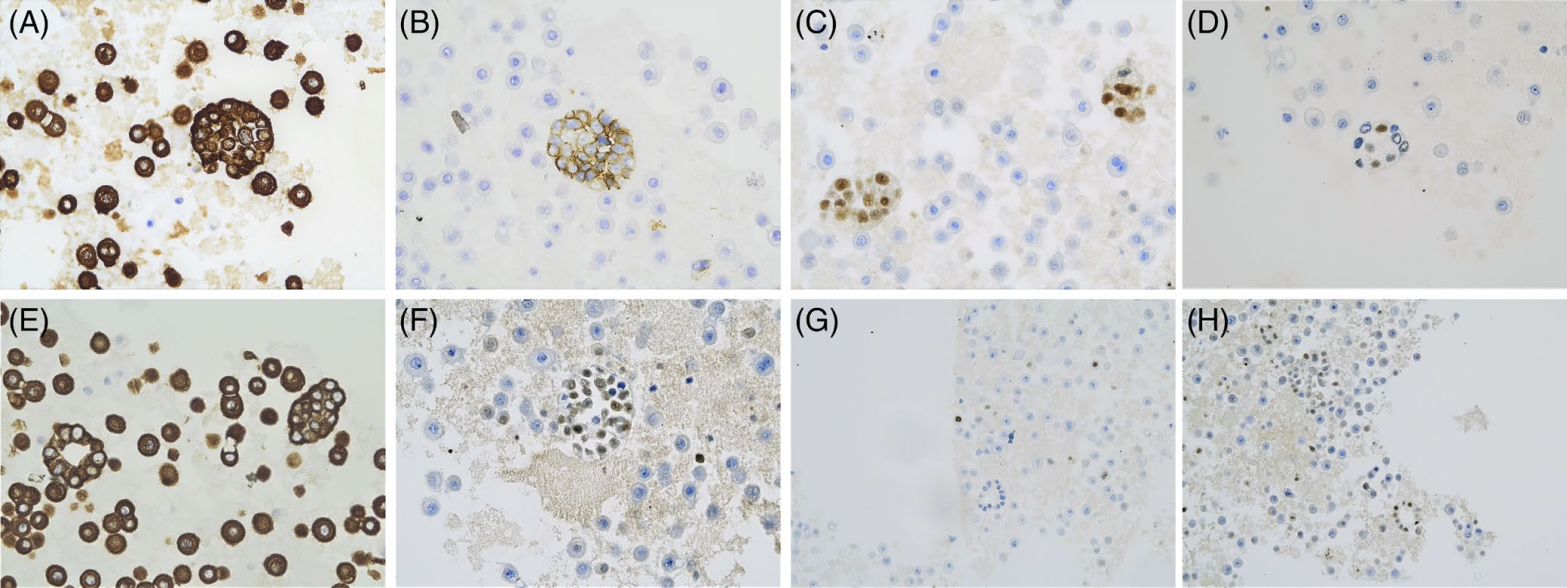
Immunohistochemical images using the cell block method. The tumor cells are positive for AE1/AE3 (A), BerEp4 (B), GATA‐3 (C), Androgen Receptor‐AR (D), Cam 5.2 (E), Estrogen Receptor‐ER (F) (400×), and Ki67 (G), Progesterone Receptor‐PR (H) markers (original magnification 200×).

Immunohistochemistry (IHC) was performed on formalin‐fixed, paraffin‐embedded (FFPE) cell blocks obtained from ThinPrep stored material (Figure 1B). 4‐μm sections were cut from the blocks and placed on a Ventana Benchmark XT (Ventana Medical Systems Inc., Tucson, AZ) for staining using a protocol that included online deparaffinization and antigen retrieval, followed by primary antibody incubation. Antigen–antibody reactions were observed using the Ventana UltraView Universal DAB Detection Kit. IHC revealed a positivity of the tumor cells for BerEp4, AE1/AE3, GATA‐3, Androgen Receptor (AR, 10%), CAM5.2, Estrogen Receptor (ER, 70%), Ki67 with a score of 10% and Progesterone Receptor (PR, 10%), negativity for TTF‐1, GCDFP‐15, HER‐2, HBME‐1, and Calretinin. Immunoreactivity is shown in Figure 2A–H.

A thorough search in PubMed has been performed in order to find all cases of MBC with pleural effusion. Only three reported cases were found. The results of our literature search are summarized in Table 1.

**TABLE 1:** Previously reported cases of pleural metastasis of breast cancer in male patients.

Author	Age	Tumor type	Location	Risk factor
Zakaria EO, 2019 [10]	84	NA	Right‐sided pleural effusion	Type II diabetes mellitus.
Karmakar S, 2013 [11]	72	Ductal Carcinoma	Bilateral pleural effusion	Type II diabetes mellitus and hypertension
Hogan K, 2020 [12]	60	Adenoid cystic carcinoma	Bilateral pleural effusion	NA
Present study, 2025	55	Ductal Carcinoma	Left‐sided pleural effusion	None

## 5 Discussion

Male breast carcinoma (MBC) is an uncommon disease which shows biologic differences from breast carcinoma in women. One of the major differences is represented by the distribution of histologic types including the evidence that 64%–87% of men with breast carcinoma have invasive ductal histology [13]. This is associated with a scant or absent lobular component in male breast. Furthermore, many male breast cancers show an ER/PR‐positive disease compared to women. The effective treatment of patients with male breast carcinoma remains a clinically challenging scenario with many unanswered questions.

The role and understanding of germline mutations with increased prevalence in MBC, such as BRCA2, may help with the identification of novel treatment options such as PARP inhibitors. Furthermore, there is a constant expression of AR in MBC associated with a relatively benign safety profile, so that AR‐targeted agents may prove beneficial as a treatment option, either as monotherapy or in combination with other agents [14].

As in female breast carcinomas, MBCs are highly sensitive to hormonal changes with a peculiar hormonal imbalance between an excess of estrogen and a deficiency of testosterone, increasing the risk of the disease.

Specifically, estrogen receptors (ER) are expressed in more than 90% and progesterone receptors (PR) are in more than 80% of male breast cases [14, 15]. Therefore, the presence of both ER and PR plays a crucial role in determining if the primary disease behind the metastatic pleural effusions is breast cancer. The presence of ER and PR does not definitely support only breast cancer, due to the evidence that other cancers, including, gynecological malignancies, can metastasize to pleural effusions. Nonetheless, in the presence of negativity for ER and PR, breast cancer cannot be ruled out as a possible malignancy. In fact, in a case like ours, additional immunohistochemical markers were performed (such as, GATA‐3, calretinin, TTF‐1) for the diagnosis of breast cancer.

The patient had no BRCA mutation and there was no family history of breast cancer. Moreover, no radiation exposure or other diseases known as risk factors such as liver disease or testicular abnormalities, or obesity were reported in this patient. As a result, the exact cause of breast cancer in this patient was unclear and risk factors of male breast cancer should be further studied in the future.

At present, the patient is under chemotherapy treatment and follow‐up is still ongoing. Effusion cytology turned out to be an accurate tool for the diagnosis of metastatic carcinomas. More studies about the clinicopathological and immunohistochemical features of male breast carcinoma should be conducted to build an evidence base supporting future treatment recommendations for this rare disease. The case taught that a possible localization of effusion is likely to be possible even in male breast cancer. A combined evaluation of cytological samples with the clinical and imaging history is crucial to make a conclusive diagnosis.

## Conflicts of Interest

The authors declare no conflicts of interest.

## Data Availability

Research data are not shared.
